# Expression Profile of Nuclear Receptors along Male Mouse Nephron Segments Reveals a Link between ERRβ and Thick Ascending Limb Function

**DOI:** 10.1371/journal.pone.0034223

**Published:** 2012-03-23

**Authors:** Halla Krid, Aude Dorison, Amel Salhi, Lydie Cheval, Gilles Crambert

**Affiliations:** UPMC Univ Paris 6/INSERM/CNRS, Centre de Recherche des Cordeliers, UMRS 872 Equipe 3 Laboratoire de Génomique, Physiologie et Physiopathologie Rénales ERL 7226, Paris, France; University of Geneva, Switzerland

## Abstract

The nuclear receptor family orchestrates many functions related to reproduction, development, metabolism, and adaptation to the circadian cycle. The majority of these receptors are expressed in the kidney, but their exact quantitative localization in this ultrastructured organ remains poorly described, making it difficult to elucidate the renal function of these receptors. In this report, using quantitative PCR on microdissected mouse renal tubules, we established a detailed quantitative expression map of nuclear receptors along the nephron. This map can serve to identify nuclear receptors with specific localization. Thus, we unexpectedly found that the estrogen-related receptor β (ERRβ) is expressed predominantly in the thick ascending limb (TAL) and, to a much lesser extent, in the distal convoluted tubules. *In vivo* treatment with an ERR inverse agonist (diethylstilbestrol) showed a link between this receptor family and the expression of the Na^+^,K^+^-2Cl^−^ cotransporter type 2 (NKCC2), and resulted in phenotype presenting some similarities with the Bartter syndrom (hypokalemia, urinary Na^+^ loss and volume contraction). Conversely, stimulation of ERRβ with a selective agonist (GSK4716) in a TAL cell line stimulated NKCC2 expression. All together, these results provide broad information regarding the renal expression of all members of the nuclear receptor family and have allowed us to identify a new regulator of ion transport in the TAL segments.

## Introduction

The kidney is a multifaceted organ with both endocrine and solute transport functions. This last aspect concerns the detoxification of the organism and the regulation of volume and ion balances. Efficiency of renal functions depends on the ability of the kidney to adequately cope with physiological demands. In addition, the kidney also adapts its functions to its physiological environment (gender, age, sexual or metabolic status, period of the day etc…). Indeed, renal functions vary during the circadian cycle [1], differ between male and female [2], are affected by chronic obesity [3], [4], and are adapted to long-term physiological situations like pregnancy [5]. This ability to “analyze” its environment requires the expression of a whole set of receptors able to sense these physiological differences or variations.

To perform these functions, the kidney is structured and organized with specialized tubular segments that may contain different cell types. This structural and functional heterogeneity originates from the first step in renal development (for review see [6]). Indeed, nephrogenesis is initiated by the interaction between ureteric bud (giving rise to the distal segments) and metanephric mesenchyma cells (leading to formation of proximal segments and glomerulus). This structural complexity renders a global investigation of kidney gene expression complex, as it requires that the analysis done at least at the tubular segment level, as performed recently [7], [8], [9], [10], [11].

The nuclear receptor (NR) gene family governs most of the response programs involved in the adaptation to the physiological environment. Even though some are qualified as “orphan” receptors, NR belong to a family of ligand-dependent transcription factors responding to small lipophilic hormones, vitamins and lipids. They are classified either based on their sequence homology [12] or on their tissue expression [13]. For many of them, their expression is dependent on the circadian cycle as pointed out recently in four important metabolic tissues or organs [14].

According to Bookout and al. [13] the kidney expresses 40 different nuclear receptors at different levels (from very low to high abundance). However, this “whole” evaluation of gene expression is not deep enough to understand the particular role of each receptor in ultrastructured organs such as the kidney. It may, for instance, hide the importance of a gene that is poorly expressed in the whole kidney but specifically expressed in a given nephron segment, which would indicate a specific regulation of particular renal functions.

To understand the involvement of NR in the regulation of the renal function, we established the quantitative expression profile of this gene family along the nephron of adult male mice. This map clearly allows for the identification of nuclear receptors with segment-specific expression. Among them, ERRβ turned to be a thick ascending limb (TAL)–specific NR, which regulates the expression of the NKCC2 transporter and thereby modulates the ability of the kidney to reabsorb sodium, potassium and water.

## Materials and Methods

### Animals and renal tubule isolation

Animal experiments were carried out according to the French legislation and performed under agreement 75–1551 (attributed to L.D.) of the Veterinary Department of the French Ministry of Agriculture. All experimental procedures involving physiological analysis of renal functions in mice were approved by the local Ethical Committee of the Cordelier research Center. Kidneys from CD1 male mice (10 weeks old, Charles Rivers Breeding Laboratories) were perfused as described previously [15]. The following structures were microdissected according to morphologic and topographic criteria: proximal convoluted tubules (PCT), proximal straight tubules (PST), medullary and cortical thick ascending limb of Henle's loop (mTAL and cTAL), distal convoluted tubule (DCT) connecting tubules (CNT), cortical and outer medullary collecting duct (CCD and OMCD). As shown recently, this manual method of tubule selection allows us to separate renal segments with a minimum of contamination, if any, of one type with another [9]. In this paper, the degree of cross contamination was evaluated to be in the range of 0.03% and 5%. Animals were sacrificed between 2 and 4 hours after the beginning of the light period in the animal facility.

### RNA extraction, reverse transcription and quantitative PCR

RNAs extracted as previously described [16] from pools of 20–30 microdissected tubules were reverse-transcribed (Roche Diagnostics, France) according to the manufacturer's instructions. Real-time PCRs were performed on a LightCycler (Roche Diagnostics) with the 480 SYBR green I Master kit (Roche Diagnostics) according to manufacturer's instructions except that the total reaction volume was reduced 2.5-fold. PCRs were performed with cDNA quantity corresponding to 0.1 mm of tubules. No DNA was detectable in samples that did not undergo reverse transcription or in blank run without cDNA. Cyclophilin expression does not vary according to the type of segment. Specific oligonucleotides were designed using the Probe Design 2,0 software (Roche). For evaluation of circadian expression, RNA samples of whole kidney harvested at 6 different times from wild-type and clock-null mice [17] were obtained from Dmitri Firsov's group (University of Lausanne). The day is divided into ZT (Zeitgeiber time) periods. ZT0 corresponds to the time when the light is turned on and ZT12 when it is turned off. For a rodent, the activity period corresponds therefore to the part of the day from ZT12 to ZT0 whereas the rest period is between ZT0 and ZT12.

### Protein homogenate and Western blot

After sacrifice, kidneys were removed, weighed and cut into small pieces. Minced tissues were then homogenized in a buffer (250 mM sucrose, 1 mM EGTA and 10 mM NaOH-Hepes, pH 7,4) containing a protease inhibitor cocktail (Roche). After a 1000× g centrifugation (10 min. at 4°C), the supernatant was centrifuged at 100000× g (Beckman, rotor 70.1 TI) for 90 min at 4°C. The pellet, containing the microsome fraction, was resuspended in the homogeneisation buffer. Protein concentration was determined using the Bradford protein assay (Bio-Rad). Fifhty µg of proteins were denaturated by 2X protein sample buffer (4,8% SDS, 6,9% sucrose, 0,012% bromophenol blue, 2,1% β-mercaptoethanol) and heated 8 min at 65°C. The samples were then resolved onto a 7,5% SDS-polyacrylamide gel and transferred onto a PVDF membrane for immunoblotting using a polyclonal anti-NKCC2 (gift from Dr. Amlal, Cincinnati, OH, USA).

### Metabolic analysis

Experiments were performed on male CD1 mice (Charles River, L'Arbresle, France) weighing 22–26 g at the beginning of experiments. Animals were fed the standard laboratory diet (Safe, France) *ad libitum* with free access to deionized water. For metabolic analysis, baseline 24-h urine volume, food and water intakes were measured after 3 days of cage adaptation. Diethylstilbestrol (DES) was injected sub-cutaneously (sc) every 24 h after solubilization in sesame oil mixture (100 µg/day/mouse) or with vehicle only for 7 days. Urinary creatinine was determined on an automatic analyzer (Konelab 20i; Thermo, Cergy Pontoise, France) and electrolyte concentrations (Na^+^ and K^+^) were determined on a flame spectrophotometer. Blood parameters were measured on anesthetized mice (pentobarbital 50 mg/kg) after retro-orbital puncture and using an ABL77 pH/blood-gaz analyzer. To test the furosemide sensitivity, control and DES-treated groups were injected i.p. with a single dose of furosemide (1 mg). After 90 min. their urines were analyzed for Na^+^ and K^+^ contents and volume. All experiments were performed in accordance with the French legislation for animal care and experimentation.

### Cell line and treatment

A previously described cell lines originating from mouse thick ascending limb (MKTAL, a generous gift from Soline Bourgeois of University of Zurich [18]) was used to test a pharmacological activator of ERRβ/γ receptors GSK4716 ([19], Sigma-Aldrich) a compound that is not suitable for in vivo use due to its metabolic instability (Bill Zuercher, personal communication). Briefly, MKTAL were grown in DMEM/F12 medium (Invitrogen) containing non-essential amino acids (1%), glutamine (4 mM), HEPES (15 mM), NaHCO_3_ (25 mM), penicillin/streptomycin (50 IU/ml) and Fetal Bovine Serum (5%). MKTAL were grown on 6-well filters (Costar 3412), and starved from fetal bovine serum for 24 h before experiments.

## Results

### Nuclear receptors in kidney

**Figure 1:**
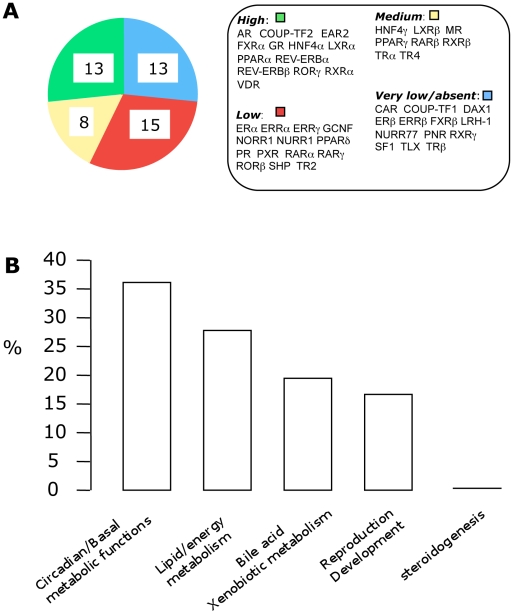
Nuclear receptor expression in the whole kidney. (A) Number of NR expressed at different level in kidney of adult male mice (n = 5). Expression levels are defined as “High” if Ct is ≤26 cycles; “Medium” if Ct is between 26 and 29; “Low” if Ct is between 29 and 32 and “Very low or absent” if Ct ≥32. (B) Renal distribution of NR according to their functional groups as defined by Bookout et al. [13].

We examined the expression of all NRs in the whole kidney of male mice. As shown in Figure 1A, 36 NRs are expressed in the kidney from a high to a low level of expression (Cp<32). Regarding the putative biological functions in which the NRs are involved (as defined by Bookout et al. [13]), we observed that NRs involved in steroidogenesis regulation (SF-1, DAX-1 and FXRβ) are absent from the kidney (Figure 1B). The group of NRs involved in the circadian and basal metabolic functions is the major one, representing 36% of all NRs expressed in the kidney; it is followed by NRs involved in lipid and energy metabolism (28%), in bile acid and xenobiotic metabolism (19%), and in reproduction and development processes (17%).

### Nuclear receptors are differentially expressed along the nephron

To probe further, we then measured the expression of NRs in the following 8 tubular segments: proximal convoluted tubule (PCT), proximal straight tubule (PST), medullary and cortical thick ascending limb (mTAL and cTAL), distal convoluted tubule (DCT), connecting tubule (CNT), cortical and outer-medullary collecting tubule (CCD and OMCD) of the kidney. The method we used to select tubules are based 1/ on their localization within kidney (cortex or medulla) and 2/ on their morphologic aspect. This method has recently allowed Cheval et al. [9] to establish the transcriptome of the different tubular segments in mouse. We first classified the NRs as a function of their abundance in each segment. For this purpose, we calculated the mean expression of all NRs in each different segment (relative to cyclophilin expression). We then compared the level of expression of a given NR in a particular segment to the mean value of all NRs in this segment (denoted by *m*). Accordingly, four groups were defined: “High” (expression level ≥2*m*); “medium” (0.5*m*≤expression level≤2*m*); “low” (0.2*m*≤expression level≤0.5*m*) and “very low or absent” (expression level≤0.2*m* or not detected).

**Figure 2:**
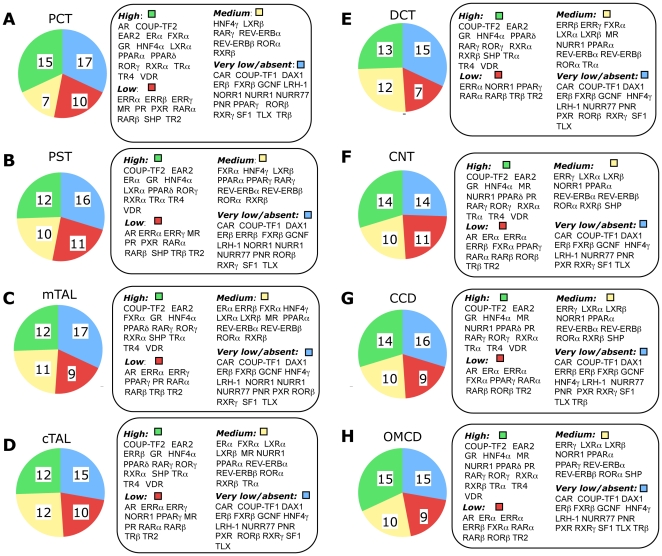
Quantitative expression of NR family gene in the different tubular segment of the nephron. Quantitative values for NR expression (relative to cyclophilin expression) were evaluated by QPCR on 0.1 mm of 8 different mouse renal segments: proximal convoluted tubules (PCT), proximal straight tubules (PST), medullary and cortical thick ascending limb of Henle's loop (mTAL and cTAL), distal convoluted tubule (DCT) connecting tubules (CNT), cortical and outer medullary collecting duct (CCD and OMCD). For each segment, the mean value of NR expression (m) was calculated and used as reference to classify the level of expression of an individual NR. “High”: NR expression level ≥2m. “Medium”: 0.5m≤NR expression level ≤2m. “Low”: 0.2m≤NR expression level ≤0.5m. Very low/absent”: NR expression level ≤0.2m or null.

As shown in Figure 2, the relative expression of the NRs in the different tubular structures of the nephron revealed that among the NRs tested, 7 are absent from all tubular segments (DAX-1, ERβ, FXRβ, NURR-77, PNR, TLX, SF-1) whereas 5 have a very low expression in many segments (CAR, COUP-TFI, GCNF, LRH-1, RXRγ). On the other hand, 10 NRs are abundantly (but sometime differentially) expressed in all segments (COUP-TF2, EAR2, GR, HNF4α, PPARδ, RORγ, RXRα, TRα, TR4 and VDR).

**Figure 3:**
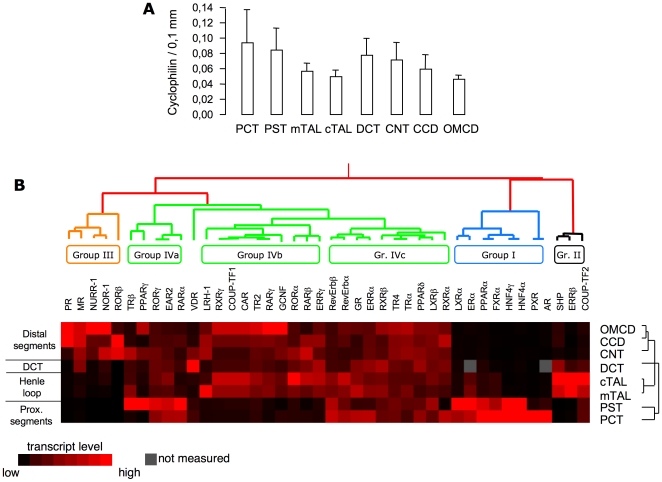
Hierarchical clustering of nuclear receptor expression profile along mouse nephron. (A) mRNA expression of cyclophilin in the different tubular segments established by QPCR. The mRNA expression profile of NR along the nephron was established by QPCR (n = 5 animals for each segment) and evaluated by unsupervised hierarchical clustering using the Cluster and Treeview software from Standford University [29].

From this simple classification it is already obvious that some NRs exhibit a more restricted expression than others. To further document this observation, we performed a hierarchical unsupervised clustering of NR expression profiles along the nephron. To avoid dispersion of our results due to possible technical artifacts (in the measurement of the tubule length or during the RNA extraction or the reverse-transcription steps) we first measured the expression of an housekeeping gene, the cyclophilin A, that will serve as reference for our analysis. As shown in Figure 3A, the expression of cyclophilin, is similar in all tubular segments. The unsupervised clustering revealed a segregation into groups whose main determinant is the nature of segments, and allowed us to identify nuclear receptors with “specific” expression independently of their overall expression level. Group I consists of 8 NRs (LXRα, ERα, PPARα, FXRα, HNF4γ, PXR and AR) that, according to the Bookout classification, predominantly belong to the lipid/energy and bile acid/xenobiotic metabolisms. Three NRs (SHP, ERRβ and COUP-TF2) display a predominant expression in the TAL segments and form group II in our classification. According to the Bookout classification, these three NRs belong to different functionality groups. Group III contains 5 NRs mainly expressed in the distal nephron tubules (PR, MR, NURR-1, NOR-1 and RORβ) that, except for PR, belong to the “basal metabolic functions” set of NR. In addition to these 3 groups whose members exhibit a clear expression in a well-defined nephron structure, a fourth, grouping the majority of the NRs, gathers the NRs that are expressed in more than one nephron structure. Group IV can be subdivided into broadly expressed NRs (Group IVa; TRβ, PPARγ, RORγ, EAR2 and RARα), NRs that are absent in proximal segments (Group IVb; LRH-1, RXRγ, COUP-TF1, CAR, TR2, RARγ, GCNF, RORα, RARβ and ERRγ) and NRs present in all segments (Group IVc; RevErbα and β, GR, ERRα, RXRβ, TR4, TRα, PPARδ, LXRβ and RXRα).

### Renal segments-specific NR expression

**Figure 4:**
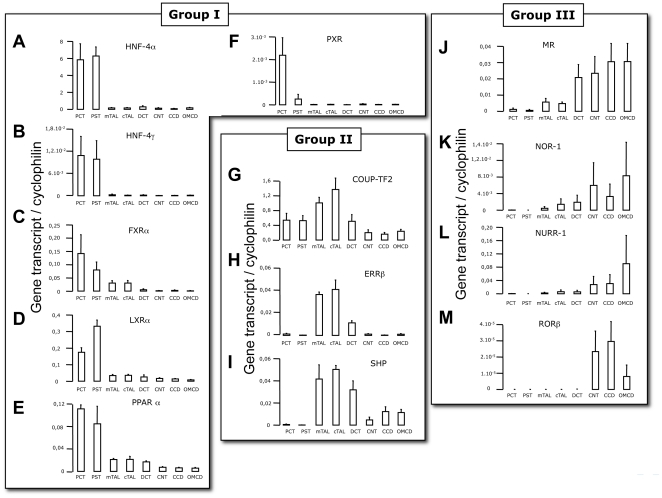
Segment-specific expression of NR. Expression of NRs in group I (proximal tubule segments, A–F), in group II (thick-ascending limb, G–I) and in group III (distal tubule segments, J–M) assayed by QPCR and divided by expression of cyclophilin. Results are shown as mean ± s.e.m. (n = 5). Test of variance among the different groups was performed (One-way ANOVA) and showed significant variability with p<0.01, for A–J and M and p<0.05 for K and L.

In group I, AR, ERα, PXR, HNF4α, HNF4γ, FXRα, PPARα display a specific expression in PCT and PST (Figure 4A–F, results for AR and ERα were published earlier [11]). Within this group, however, we notice differences both in the level and the degree of restriction of their expression. Four of them, HNF4α, HNF4γ, LXRα and PPARα (Figure 4 A, B, D and E) are highly expressed in the two segments (PCT and PST) forming the proximal tubules. FXRα (Figure 4C) has a predominant but not exclusive expression in this part of the nephron. Conversely, PXR is strictly restricted to the first part of the proximal tubule (Figure 4F). Three NRs expressed in the TAL also differ in terms of expression and restriction levels. COUP-TF2 and SHP (Figure 4G and I) are predominant in, but not restricted to, TAL whereas ERRβ (Figure 4H) is mainly expressed in both cortical and medullary TAL. In the distal part of the nephron 5 NRs (MR, NOR-1, NURR-1, RORβ and PR, Figure 4J–M) are expressed in the CNT, CCD and OMCD (PR expression along the nephron was published earlier [11]). Here again, some differences may be outlined within this group in terms of restriction and expression level. The mineralocorticoid receptor (MR, Figure 4J) is expressed from the DCT to the OMCD whereas the progesterone receptor (not shown here, see [11]) is more restricted to the CCD and to OMCD.

### Characteristics of renal ERRβ expression: a rhythmic TAL-specific NR

**Figure 5:**
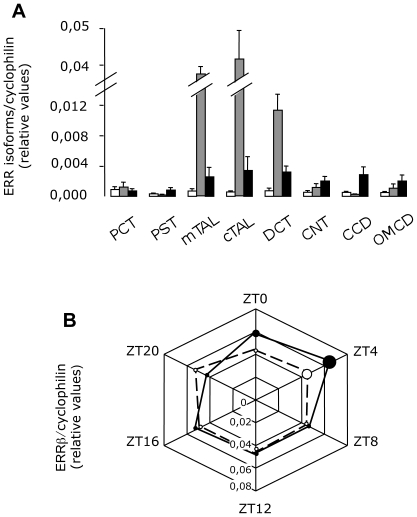
Characterization of ERR family in the kidney. Quantitative expression of ERRα (white bars), ERRβ (grey bars) and ERRγ (black bars) along the nephron (A). Results are shown as mean ± s.e.m (n = 5). Circadian expression of ERRβ in wild-type (plain line) and in clock-null mice (hatched line). Results are shown as the mean ± s.e.m. (n = 6). The size of the dots is proportional to the s.e.m. values. Test of variance among the different groups was performed (One-way ANOVA) and showed significant variability in wild-type mice (p<0.01) but not in clock-null mice.

It is therefore a total of 15 NRs that exhibit a restricted expression along the nephron. Among these segment-specific NRs, we identified ERRβ as a NR specific of the TAL. This receptor is not only specific but also quantitatively the most abundant ERR isoform in these segments (Figure 5A). Interestingly, the expression of this gene has been shown to be strongly dependent on the circadian rhythm in different tissues such as the brown and white adipose tissues, skeletal muscle, and the liver [14]. As shown in the figure 4B (plain line), the expression of ERRβ also exhibits a circadian variation in the kidney with a peak value at ZT4. This variation is clearly linked to the internal clock mechanism since clock null-mice loose this cyclic expression (Figure 5B, dotted line).

### ERRβ modulates TAL function

**Figure 6:**
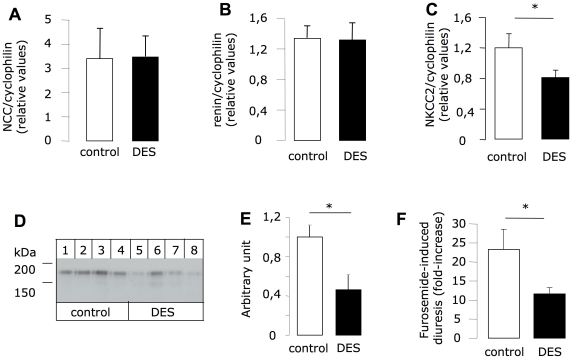
Consequences of ERR inhibition on TAL functions. After seven days of treatment with DES (100 µg/mouse)/day or vehicle, the mRNA level of NCC (A), renin (B) and NKCC2 (C) were assessed on whole kidney RNA extracts. NKCC2 protein level were evaluated by Western Blot (D and E) from kidney homogenates of control or DES-treated mice. (F) Following the same protocol of DES treatment, furosemide was injected in all mice at day 7 (DES or control groups) and urine was collected on a period of 90 minutes. Results are shown as the mean±s.e.m (n = 9). Non-paired Student t-test, * p<0.05.

To investigate the possible function of ERRβ in the TAL, we treated mice with diethylstilbestrol (DES), a pan-ERR inhibitor [20] since there is no specific inhibitor for ERRβ. After a week of treatment (Table 1), urinary volume was significantly (p = 0.008) increased by 70%, and was accompanied by a 50% decrease of urine osmolarity (p = 0.036). This urinary concentration defect could be due to a vasopressin deficit or to a TAL dysfunction. However, the DES-induced increase in hematocrit (p = 0.034), corresponding to a contraction of the extracellular volume, is more likely the consequence of a fluid leak due to inhibition of TAL ion transport. This treatment also led to a greater urinary Na^+^ excretion (34%) and a very significant increase in the urinary Na^+^/K^+^ ratio. The plasma K^+^ was slightly but significantly decreased (by 10%) whereas other measured plasma parameters, such as calcium and biarbonate levels, were not altered. We did not observed variation in urinary calcium excretion. These results suggest an effect on the expression of TAL- or DCT-specific genes. As shown in Figure 6, a 7-day treatment with DES neither affects NaCl-cotransporter (NCC) expression (specific of the DCT; A) nor renin expression (specific of the macula densa cells; B). Conversely, this treatment significantly decreased mRNA level of the Na-K-2Cl cotransporter (NKCC2; Figure 6C). This observation is confirmed at the protein level (Figure 6D and E). When we tested the in vivo activity of NKCC2 by measuring the sensitivity to furosemide of control and DES-treated mice, we also confirmed a decrease in NKCC2 function in the latter. Indeed, furosemide increased urinary volume by a factor of 22 in the control group, and by a factor of 12 in the group treated with DES (Figure 6D).

**Table 1:** Physiological parameters of mice treated or not with diethylstilbestrol for 7 days.

*Urine parameters*			
	Control	DES 7 days	*p* values
Volume (ml)	1.3±0.3	2.2±0.3	*0.008*
Osmolarity (mosm/kgH20)	2654±489	1279±288	*0.036*
Na^+^ excretion (mmol/mmol creat)	39.6±3.1	53.2±4.9	*0.039*
K^+^ (excretion (mmol/mmol creat)	23.3±1.7	20.8±2.3	0.4
Na^+^/K^+^ ratio	1.78±0.07	2.63±0.16	*>0.001*
Ca^2+^ (excretion (mmol/mmol creat)	0.4±0.1	0.2±0.05	0.086

*Plasma parameters*			
	Control	DES 7 days	*p* values
Hematocrit (%)	38.1±0.4	40.7±1.0	*0.034*
Plasma Na^+^ (mM)	147.9±0.5	148.9±0.6	0.24
Plasma K^+^ (mM)	4.0±0.2	3.6±0.1	*0.041*
pH	7.22±0.02	7.22±0.03	0.92
Bicarbonate (mM)	21.7±0.8	21.0±0.7	0.53
Plasma Ca^2+^ (mM)	1.24±0.01	1.26±0.01	0.31

Results are shown as mean ± s.e.m. (n = 9). Non-paired Student t-test was used for comparing set of data.

**Figure 7:**
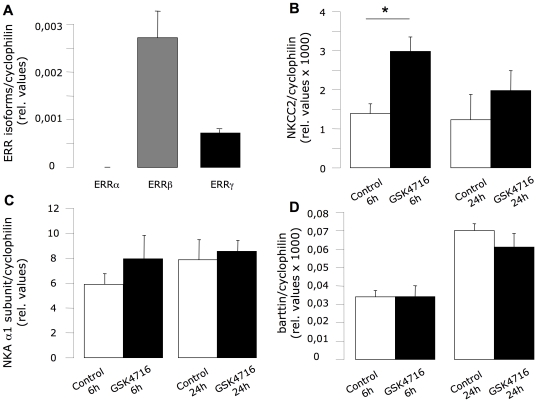
Consequences of ERRβ/γ activation on NKCC2 expression. (A) MKTAL cell line was grown on filters for 5 days before 24 h serum starvation and used for measuring expression of ERR isoforms (ERRα white bars; ERRβ grey bars, ERRγ black bars) by QPCR. Results are shown as the mean±s.e.m (n = 9). Test of variance among the different groups was performed (One-way ANOVA) and showed significant variability (p<0.01). After the starvation period, cells were treated with 10 µM of GSK4716 (Sigma-Aldrich) or vehicle (DMSO, 0,1%) for 6 or 24 h and the expression levels of NKCC2 (B), of Na,K-ATPase α1 subunit (C) and of Barttin (D) were then tested by QPCR. Results are shown as the mean±s.e.m (n = 9). Non-paired Student t-test, * p<0.05.

Because the ERRβ agonist GSK4716 is not suitable for in vivo experiments, we used a cell line model of the TAL segment developed few years ago by Bourgeois et al. [18]. This mouse cell line (MKTAL) has a similar ERR isoform expression pattern compared with native TAL cells, with ERRβ being the preponderant form (Figure 7A). As shown in Figure 7B, a 6h-period incubation of these cells with GSK4716 increased the expression of NKCC2 by a factor of 2. On the contrary, neither the expression of the Na,K-ATPase α1 subunit (Figure 7C) nor that of the barttin (Figure 7D) is affected by incubation with GSK4716.

## Discussion

### Differential expression of NR along the nephron: What does it teach us?

Our results show the necessity not only to explore gene expression patterns at the whole organ level but also to take into account the structural complexity of organs. Indeed, our study shows that the presence at high or low expression levels in the whole kidney may mask more subtle differences. For instance, half of NRs appeared to be absent or expressed at low levels in the kidney; however the segmental analysis revealed that some of them are actually specific to restricted portions of the nephron (ERRβ, TRβ, ERα, PR, PXR, RORβ, SHP, PXR, PR). Conversely, a gene highly expressed at the whole organ level may only display a specific expression in an abundant structure. For instance, because the relative mass of proximal tubules is much greater than that of other kidney segments, some NRs exhibiting a strong expression in the whole kidney are actually specifically expressed only in PCT and/or PST (AR, FXRα, HNF4α, LXRα, PPARα). The knowledge of the exact renal localization of NR is therefore critical to decipher their physiological relevance for kidney function. Our qPCR results are in good agreement with published results that localized the PPAR isoforms along the rat nephron [21] and showed, for instance, the restricted expression of PPARα in the proximal tubules and a rather broad expression of RXRα and β receptors. Similarly, COUP-TFII has been shown to be highly expressed in cortical structure that are not PCT/PST or CNT/CCD [22] and which could be either the cortical TAL or the DCT.

Analysis of the hierarchical clustering of NR expression in the nephron failed to display a clusterization that depends on the known or putative functions of NR as defined by Bookout et al. [13]. The main factor that governs the distribution of NRs is the nature of the segments. However, some NR functions are more represented in some segments than in others. For instance, group I (proximal segments) expresses a high number of NR involved in the regulation of “nutrient metabolism”. In fact, all PT-specific NRs (except AR and ERα) belong to the group “nutrient metabolism” and more predominantly to its subdivision “lipid and energy metabolism”. This is in good agreement with the strong activity of solute and protein transport that occurs in this initial part of the nephron, which requires energy consumption.

One conclusion of the study published by Bookout et al. [13] was that NRs that display similar tissue expression profiles are governed by a common mechanism for their transcriptional regulation. Our investigation within a particular organ revealed however a more complicated scheme. For instance, at the tissue level, HNF4α and VDR display a similar pattern in the so-called gastroenteric tissues, but within the kidney, these two NRs exhibit a distinct expression profile indicating that their expression is subject to different regulatory mechanisms. Conversely, we showed in this study the close similarity in tubular expression between ERRβ and SHP whereas these two NRs are clearly dissociated at the tissue level.

### Role of ERR in the kidney

One of our observations is the predominant expression of the β isoform of the ERR receptors in the thick ascending limb of the Henle loop. This segment contributes significantly to renal Na^+^ and K^+^ reabsorption, and in parallel to the constitution of the medullo-papillary osmotic gradient because it is water-impermeable. TAL dysfunction leads to increased diuresis, urinary loss of Na^+^ and to possible variations in blood pressure and plasma K^+^ values, as is the case with Bartter's syndrome. The presence of ERRβ in this part of the nephron may therefore be of particular interest. There is recent evidence that ERR isoforms (α and γ) have a direct impact on Na^+^ and K^+^ homeostasis. Tremblay et al. [23] showed that ERRα knock-out mice display strong blood pressure perturbations that are related to renal dysfunction. Interestingly, in this study, Tremblay et al. identified genes that are directly regulated by ERRα through binding of this NR close to their genomic region. Among which different ion transporters are found (the constituting subunits of the Na,K-ATPase, K^+^ channels and other solute transporters). On the other hand, absence of ERRα also leads to dysregulation of the expression of genes such as NKCC2 but the CHiP-on-chip data do not reveal a direct interaction between ERRα and NKCC2 promoter region.

As for ERRγ, the phenotype analysis of the knock-out mice (on newborns) revealed a link between this isoform and the maintenance of Na^+^ and K^+^ homeostasis [24]. However, in these two studies it was not possible to clearly distinguish the effects that are directly due to renal dysfunction from those related to systemic modifications.

Here, we showed that ERRβ is one of the NR exhibiting a segment-specific expression along the nephron. Because of fetal death due to malformation of the placenta [25], there is no ERRβ knock-out mouse model to establish the complete renal phenotype. However, a genetic mouse model has been developed in which ERRβ is specifically deleted in the inner ear [26]. This study clearly revealed that ERRβ governs the expression of multiple ion transporters including NKCC1 and the Na,K-ATPase α1 isoform. A related finding indicates that patients suffering from a non-syndromic hearing impairment had specific missense mutations of the ERRβ coding gene [27]. We speculated therefore that ERRβ could also modulate the expression of different ion transporters in the TAL segments. Our results showed that in vivo inhibition of ERR affects indeed whole body fluid and ion homeostasis, that it impacts TAL function and more particularly the expression of NKCC2. An effect related to estrogen receptors (ER) effect and mediated by the DES is rather unlikely since estradiol promotes the expression of NKCC2 [28]. However, because of its multiple action, we cannot only relate the renal action of DES to inhibition of ERRβ. It is, however, obvious that the broad action of DES on the three ERR isoforms and on ER We therefore used a more direct approach involving an ERRβ agonist GSK4716 that is not suitable for in vivo experiments and a cell line derived from the mouse TAL. Under these conditions, activation of ERRβ resulted in an increased expression of NKCC2 but does not affect expression of the Na,K-ATPase or of the Barttin.

Since ERRβ is described as a constitutively active receptor because of the constant presence of a lipid molecule docked in its ligand-binding pocket, its activity should depend on its own expression level and on the presence of different co-regulators. Here, we described one physiological situation, the adaptation to the circadian rhythm, where ERRβ expression levels are modulated. The consequences of this circadian variation need to be investigated further, but based on our observations regarding ERRβ renal function, it is possible that it participates in the diuresis circadian variations. Another possibly relevant aspect of the TAL-specific expression of ERRβ relates to Bartter's syndrome. A number of patients exhibiting the phenotype of this syndrome do not display genetic polymorphisms on the coding sequence of the four related genes (NKCC2, ROMK, CLC-K and Barttin). The new putative regulator of TAL function that we have identified may provide some insight into the origin of as-of-yet unexplained Bartter's syndrome phenotypes.

All together, these results demonstrate the importance of analyzing gene distribution at the level of the structures that constitute an organ, in order to reveal the specificity and physiological relevance of the corresponding proteins. This strategy led to the finding of a TAL-specific NR, ERRβ, which may be involved in the regulation of NKCC2, and more generally, of ion and solute balance. Further studies should explore the renal NR expression map more in depth and, regarding ERRβ more specifically, seek to understand 1/ under which physiological conditions this regulation actually occurs and 2/ the consequences of inhibiting this regulatory pathway.
